# The effects of zinc supplementation on the metabolic factors in patients with non-alcoholic fatty liver disease: a randomized, double-blinded, placebo-controlled clinical trial

**DOI:** 10.1186/s40795-023-00776-z

**Published:** 2023-11-27

**Authors:** Seyed Mohammad Amin Rezaei, Farzaneh Mohammadi, Mohammad Hassan Eftekhari, Fardad Ejtehadi, Haleh Ghaem, Nazanin Mohammadipoor

**Affiliations:** 1 Department of Clinical Nutrition, School of Nutrition and Food Sciences, Shiraz University of Medical Sciences, Shiraz, Iran; 2 Student Research Committee, Shiraz University of Medical Sciences, Shiraz, Iran; 3 Gastroenterohepatology Research Center, Shiraz University of Medical Sciences, Shiraz, Iran; 4 Non-Communicable Diseases Research Center, Department of Epidemiology, School of Health, Shiraz University of Medical Sciences, Shiraz, Iran

**Keywords:** Non-alcoholic fatty Liver Disease, Zinc, Aminotransferases, Metabolic factors

## Abstract

**Background:**

Non-alcoholic fatty liver disease (NAFLD) is associated with metabolic factors including obesity, dyslipidemia, insulin resistance, oxidative stress, and elevated inflammatory factors. Zinc (Zn) supplementation has been investigated as a potential adjunctive therapy in managing NAFLD outcomes.

**Methods:**

In this randomized, double-blinded, controlled clinical trial, 50 overweight or obese participants with NAFLD were randomized into 2 groups of 25 and received either 30 mg of daily Zn or a placebo for 8 weeks. Both groups were invited to follow a balanced energy-restricted diet and physical activity recommendations.

**Results:**

Based on the between-group comparison, Zn supplementation caused a significant increase in the Zn level (P < 0.001) and a significant decrease in weight (P = 0.004), body mass index (BMI) (P = 0.002), waist circumference (P = 0.010), aspartate transaminase (AST) (P = 0.033), total cholesterol (TC) (P = 0.045), and low-density lipoprotein cholesterol (LDL-C) (P = 0.014), but it had no significant effect on alanine transaminase (ALT), fasting blood sugar (FBS), insulin, homeostasis model assessment of insulin resistance (HOMA-IR), high-density lipoprotein (HDL), triglyceride (TG), high-sensitivity C-reactive protein (hs-CRP), malondialdehyde (MDA), and total antioxidant capacity (TAC) (P > 0.05).

**Conclusion:**

The results of the present study indicated that 8-week supplementation of 30 mg daily Zn may increase the Zn serum level and decline anthropometric parameters, AST, TC, and LDL-C in NAFLD patients, so further research is suggested in the future.

**Trial registration:**

The trial was retrospectively registered at IRCT.ir as IRCT20191015045113N1 (December/8/2019).

## Introduction

Non-alcoholic fatty liver disease (NAFLD) is one of the most common liver diseases which develop in response to accumulation of fat (mostly triglyceride (TG)) in the hepatocytes [1, 2]. The worldwide prevalence of NAFLD is about 20–30%, and the NAFLD prevalence was about 33.9% in 2016, in Iran [3, 4]. NAFLD is associated with metabolic factors including obesity, dyslipidemia, insulin resistance (IR), oxidative stress, and elevated inflammatory markers. It also increases the risk of developing diabetes mellitus (DM) and cardiovascular diseases (CVD) [5]. The first-line therapy for NAFLD is lifestyle modification and exercise. Given the poor compliance with lifestyle modification, some studies indicate that a combination of these measures with pharmacological interventions and dietary supplementation would have more favorable effects [1, 6]. Zinc (Zn), an essential trace element, involves the enzymatic activities and structural maintenance of numerous enzymes and proteins, and it has various physiological roles in the body. Based on other studies, Zn deficiency in the body is associated with diseases such as metabolic syndrome, DM, and chronic liver diseases. Zn supplementation effects have been proven to improve IR and oxidative stress; reduce total cholesterol (TC), TG, and low-density lipoprotein cholesterol (LDL-C); and decrease some pro-inflammatory factors in the body [7–9]. According to our knowledge, there has been no study in Iran investigating the effects of Zn on metabolic factors in patients with NAFLD. Thus, the aim of this study was to evaluate the effect of Zn supplementation on the serum Zn status, liver aminotransferases, anthropometric indices, and metabolic factors such as glycemic indices, lipid profile, inflammatory factors, and oxidative stress in NAFLD patients.

## Methods and materials

### Study design and participants

**Fig. 1:**
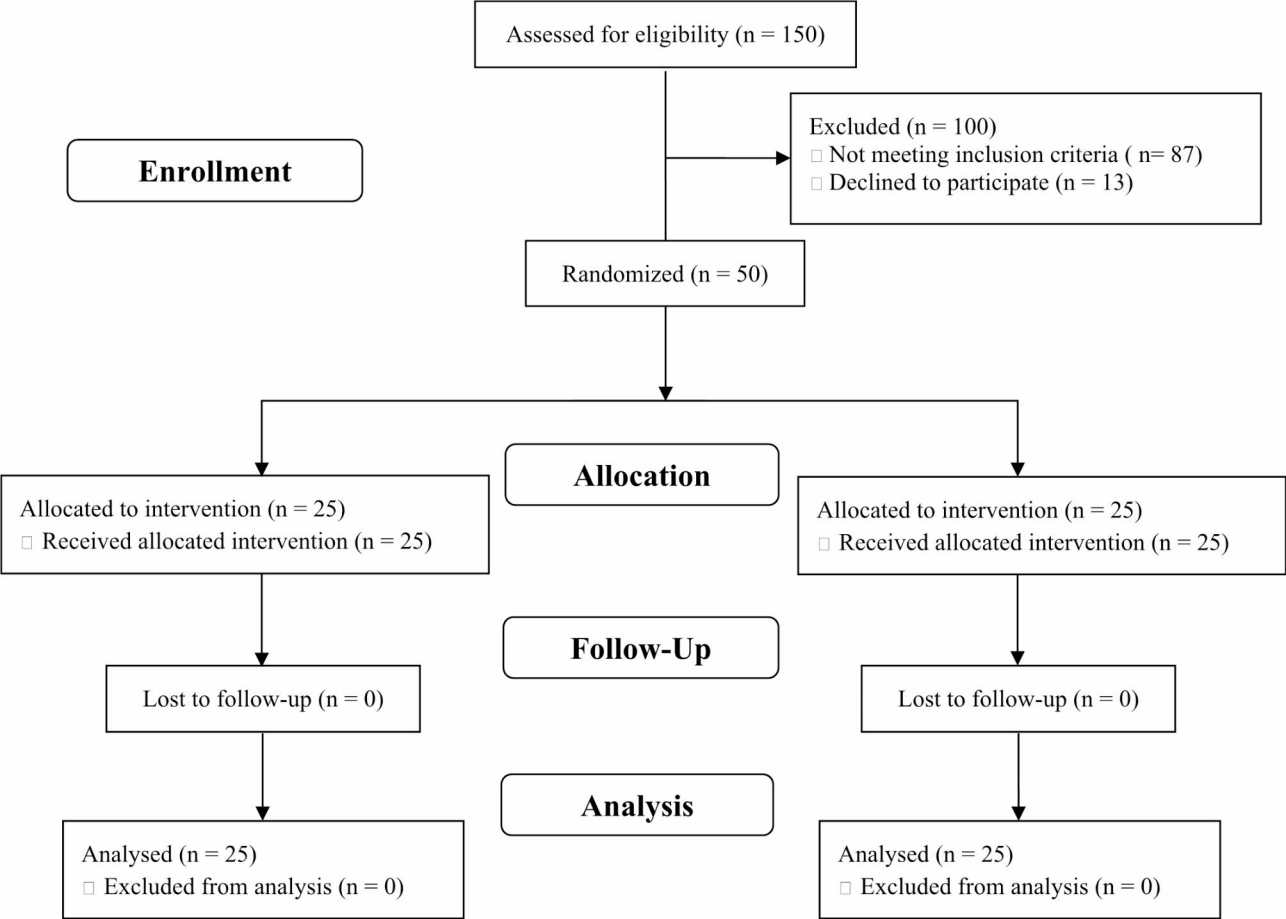
CONSORT flow chart of the study

The study protocol of this 8-week randomized, double-blinded, controlled clinical trial was approved by the Ethics Committee of Shiraz University of Medical Sciences, Shiraz, Iran (IR.SUMS.REC.1397.105) and registered at the Iranian Registry of Clinical Trials (IRCT20191015045113N1). This study was also performed based on the declaration of Helsinki and good clinical practice guidelines from September 2018 to September 2019 at Imam Reza and Motahari Clinics, Shiraz, Iran. Participants were recruited for the study through a newspaper ad and posters at Motahari and Imam Reza clinics, supervised by a gastroenterologist. The sample size was estimated 25 people in each group, based on the variation in the serum zinc in the previous study [10] with a power test of 80%, a significance level of 5%, and a dropout rate of 10%. A simple random allocation method based on the random table number was used to allocate the participants into intervention and control groups. Of the 150 patients screened, only 50 met the inclusion criteria and were randomly divided into two groups of 25 subjects (Fig. 1: CONSORT flow chart of the study). Inclusion criteria were overweight or obese individuals with ultrasound-confirmed NAFLD, age 18 to 70 years (male and female), no alcohol consumption, no liver cirrhosis, viral hepatitis, hepatocellular carcinoma, Wilson^’^s disease, acute fatty liver of pregnancy, history of chronic liver disease, no lipodystrophy, lack of parenteral nutrition, no diseases of the bile ducts, no severe weight loss in the last 6 months, no congenital metabolic diseases, no pregnancy or lactation, no drugs that cause fatty liver (methotrexate, tamoxifen, Valproate, etc.), no serum alanine transaminase (ALT) level more than 10 times the permissible limit, no history of severe systemic diseases such as CVD and kidney disease, no chemotherapy during the previous year, no alcohol and drug poisoning, and lack of consumption of supplements containing Zn. Exclusion criteria included severe allergies and side effects due to taking supplements, unwillingness to continue the study, non-compliance with the recommendations and diet provided, and consumption of less than 90% of the supplements offered. The included patients were who eat recommended 5 meals per day, substitute simple carbohydrates with complex carbohydrates, decrease refined grains intake and replace them with vegetables and fruits, decrease fructose sweeteners intake, and do at least 20 min of physical activity per day for a 2-week run-in period before the beginning of the study. After this run-in period, the intervention group received a dietary plan plus 30 mg Zn daily by zinc gluconate capsule (Nature Made, USA), while the control group received a dietary plan plus 30 mg starch powder (placebo) daily. For blinding, a capsule containing an ineffective powder (starch) completely similar to the Zn capsule was used in terms of shape, color, and size. Given that the recommended dietary allowance (RDA) and the tolerable upper intake level (UL) of the Zn in adults are 8–11 mg/day and 40 mg/day, respectively, as well as the absence of any side effects by the dose of 30 mg/day in the past studies [10, 11], this dose was selected for the present study. The diet contained a limit of 500–1000 kcal/day of estimated energy requirement, 50–55% carbohydrates, 15–20% protein, and 30% fat. The study was double-blinded for patients and physicians. Only the principal investigator could decode the contents of each capsule based on the original form of randomized results. The participants were divided into two equal groups of intervention and control based on a random number table. Demographic information and informed consent were obtained from participants at the beginning of the study. Serum Zn status, liver aminotransferases, anthropometric indices, dietary intake, physical activity, and metabolic factors such as glycemic indices, lipid profile, inflammatory factors, and oxidative stress were assessed at the beginning and end of the study.

### Compliance assessment

Once every two weeks, patients were followed up by telephone to remind them of capsule use, record side effects, and reduce the dropout rate. At the end of the fourth week, patients received the rest of the capsules from the clinic for the following weeks. Adherence to the study was ascertained by counting the remaining capsules. If the remaining capsule count of each patient was more than 10% of the expected count, the patient was excluded from the study.

### Dietary intake assessment

The patients’ dietary intakes were evaluated by a 3-day food record questionnaire (2 regular days and one weekend day). Then, the Nutritionist 4 software (First Databank Inc., San Bruno, CA, USA), modified for Iranian foods, was used to analyze these records, and total energy, macronutrients, fiber, and Zn intakes.

### Physical activity assessment

Physical activity was measured by a 7-item International Physical Activity Questionnaire (IPAQ) about the severity and duration of physical activity over the previous week. The metabolic equivalent (MET) determined 3.3, 4, and 8, for light (walking), moderate (Carrying light load, bicycling at a regular pace, and volleyball), and vigorous (Heavy lifting, digging, aerobic or fast bicycling, soccer, and running) activities, respectively. Then, physical activity was calculated by multiplying the intensity (MET) of physical activity by the duration (minute) of physical activity (MET·min/week).

### Anthropometric assessment

Height was measured while standing, without shoes, using a tape measure to the nearest 0.5 cm. Weight was measured in light clothing, without shoes by a scale (Seca, Germany) to the nearest 100 g. BMI was calculated by using the following formula: BMI = weight (kg)/ height^2^ (m^2^). Waist circumference was measured with a tape measure horizontal to the ground, between the last rib and the iliac crest with an accuracy of 1 cm.

### Biochemical assessment

Five cc blood sample was collected after 10–12 h of fasting at the beginning and end of the study at the Motahari Clinic laboratory, Shiraz, Iran. The samples were centrifuged at 4000 rpm for 10 min and stored in a freezer (-70 °C) at the laboratory of the Faculty of Nutrition and Food Sciences of Shiraz University of Medical Sciences, Shiraz, Iran for further analysis. Serum insulin and hs-CRP levels were determined by the enzyme-linked immunosorbent assay (ELISA) method through a kit (Crystal D, China). TC, TG, LDL-C, high-density lipoprotein cholesterol (HDL-C), ALT, aspartate transaminase (AST), and fasting blood sugar (FBS) were measured using standard enzymatic methods through commercial kits (Pars Azmoun, Iran) and calorimetry (auto-analyzer BT-1500, Italy). Malondialdehyde (MDA) was measured by thiobarbituric acid reactive substances (TBARS), using a spectrophotometric assay through a PD-303 spectrometer (Apel, Japan). Total antioxidant capacity (TAC) was measured using the ferric-reducing ability of plasma (FRAP) method through a kit (Zell Bio, Germany). IR also was determined by the homeostasis model assessment of insulin resistance (HOMA-IR) using the following formula: fasting insulin (µU/L) x fasting glucose (nmol/L)/22.5. Colorimetric spectrophotometry assay was used to measure the serum Zn.

### Statistical analysis

Statistical Package for Social Sciences (SPSS) software (version 19.0, SPSS Inc., Chicago, IL, USA) was used to perform the data analysis. The significance level was considered P < 0.05. Data were stated as mean ± standard deviation (SD). The normality of data distribution was assessed by the Shapiro-Wilk test. The homogeneity of qualitative variables between the groups was tested by the Chi-square test. Paired t-test and Independent Sample t-test were applied for within-group and between-group comparisons of the variables, respectively. Moreover, for adjusting confounder variables, analysis of covariance (ANCOVA) was used.

## Results

Out of 150 participants under screening, 100 were excluded from the study due to a lack of inclusion criteria (n = 87) and lack of cooperation (n = 13). Fifty NAFLD patients who met the inclusion criteria were randomly allocated into the intervention and control groups. These individuals completed the study and were analyzed (Fig. 1. CONSORT flow chart of the study). The participants’ mean age was 44.68 ± 9.90. No significant differences were observed in terms of demographic variables among the subjects (Table 1). There was no significant difference in dietary intake and physical activity, except for carbohydrates (P = 0.034) at the beginning of the study between the participants. Although the mean differences for dietary intakes of energy, protein, fiber, Zn, and physical activity were not significant (P > 0.05), the mean differences in carbohydrate (P = 0.011) and fat (P < 0.001) intakes they were (Table 2). Regarding the anthropometric variables, no significant difference was indicated between the two groups at the beginning of the study. Weight, BMI, and waist circumference decreased significantly in both groups (P < 0.001). In addition, the decreased mean differences of these variables were significant after adjusting for baseline HDL-C, fat, and carbohydrate (P < 0.05) (Table 3). No significant difference was observed in the baseline of biochemical parameters, except for HDL-C between the groups. Within-group changes in Zn (P < 0.001), ALT (P = 0.013), AST (P = 0.030), FBS (P = 0.033), Insulin (P < 0.001), HOMA-IR (P < 0.001), TC (P = 0.03), LDL-C (P < 0.001), HDL-C (P = 0.019), and TG (P = 0.001) variables were significant in the intervention group, but not in the control group. Within-group changes in hs-CRP, MDA, and TAC were significant in the neither intervention group nor the control group. However, the mean difference of Zn (P < 0.001), AST (P = 0.033), TC (P = 0.045), and LDL-C (P = 0.014) between the two study groups was significant; this value was not significant for ALT, FBS, insulin, HOMA-IR, HDL-C, TG, hs-CRP, MDA, and TAC variables after adjusting for baseline HDL-C, fat, and carbohydrate (Table 4).

**Table 1:** Demographic characteristics of the participants

		Total (n = 50)	Intervention (n = 25)	Control (n = 25)		P-value^*^
Age (mean ± SD)		44.68 ± 9.90	45.2 ± 8.78	44.24 ± 11.08		0.757
Gender n (%)	Male	23 (46.0)	12 (48.0)	11 (44.0)		1
	Female	27 (54.0)	13 (52.0)	14 (56.0)		
Education n (%)	Diploma or lower	32 (64.0)	15 (60.0)	17 (68.0)		0.769
	Bachelor or higher	18 (36.0)	10 (40.0)	8 (32.0)		
Occupation n (%)	Employed	30 (60.0)	15 (60.0)	15 (60.0)		1
	Unemployed	20 (40.0)	10 (40.0)	10 (40.0)		

^*^ P-value for age is measured using independent sample t-test, while for other variables chi square test was used

P-values less than 0.05 considered significant

**Table 2:** Dietary intake and physical activity of the intervention (n = 25) and control (n = 25) groups before and after the study, as well as their differences

		Before	After	P-value^#^	Mean difference
Energy (kcal/day)	Intervention	3274.28 ± 596.35	1963.08 ± 254.28	< 0.001	− 1311.20 ± 577.19
	Control	3334.40 ± 608.12	2078.80 ± 321.75	< 0.001	− 1255.60 ± 704.20
	P-value^*^	0.726	0.165		0.761
Protein (g/day)	Intervention	106.89 ± 21.13	90.32 ± 19.17	0.013	− 16.57 ± 30.76
	Control	114.97 ± 33.05	90.00 ± 17.86	0.002	− 24.96 ± 35.05
	P-value^*^	0.310	0.952		0.373
Carbohydrate (g/day)	Intervention	412.14 ± 106.88	303.60 ± 66.30	< 0.001	− 108.54 ± 109.62
	Control	477.42 ± 105.12	277.96 ± 65.64	< 0.001	− 199.46 ± 133.75
	P-value^*^	0.034	0.176		0.011
Fat (g/day)	Intervention	133.88 ± 45.25	56.45 ± 37.18	< 0.001	− 77.43 ± 40.23
	Control	109.34 ± 44.43	77.06 ± 40.83	< 0.001	− 32.27 ± 35.03
	P-value^*^	0.059	0.068		< 0.001
Fiber (g/day)	Intervention	10.96 ± 1.19	11.67 ± 0.97	0.008	0.70 ± 1.22
	Control	10.36 ± 1.49	10.96 ± 1.54	0.096	0.59 ± 1.72
	P-value^*^	0.123	0.058		0.799
Zinc (mg)	Intervention	13.20 ± 2.96	11.01 ± 1.87	0.002	− 2.19 ± 3.20
	Control	13.72 ± 5.06	10.95 ± 2.28	0.020	− 2.77 ± 5.58
	P-value^*^	0.662	0.912		0.653
Physical activity (MET*min/week)	Intervention	594.72 ± 51.48	743.16 ± 49.88	< 0.001	148.44 ± 72.39
	Control	598.36 ± 55.77	716.36 ± 68.36	< 0.001	118.00 ± 74.20
	P-value^*^	0.812	0.120		0.149

Data are reported as mean ± SD

* Independent Sample t-test

^#^ Paired t-test

P-value less than 0.05 considered significant

**Table 3:** Anthropometrics assessments of the intervention (n = 25) and control (n = 25) groups before and after the study, as well as their differences

		Before	After	P-value^#^	Mean difference
Height (cm)	Intervention	169.36 ± 8.03			
	Control	169.68 ± 9.68			
	P-value^*^	0.899			
Weight (kg)	Intervention	87.68 ± 13.76	82.08 ± 12.20	< 0.001	-5.60 ± 2.69
	Control	84.24 ± 15.49	81.08 ± 15.26	< 0.001	-3.16 ± 1.62
	P-value^*^	0.411	0.799		0.004^†^
BMI (kg/m^2^)	Intervention	30.55 ± 4.19	28.58 ± 3.52	< 0.001	-1.97 ± 0.96
	Control	28.95 ± 3.20	27.91 ± 3.15	< 0.001	-1.04 ± 0.57
	P-value^*^	0.137	0.483		0.002^†^
Waist circumference (cm)	Intervention	94.80 ± 9.92	90.08 ± 8.63	< 0.001	-4.72 ± 3.24
	Control	91.32 ± 10.24	89.29 ± 9.89	< 0.001	-2.12 ± 1.78
	P-value^*^	0.228	0.739		0.010^†^

BMI: body mass index

Data are reported as mean ± SD

* Independent Sample t-test

^#^ Paired t-test

P-value less than 0.05 considered significant

^†^ ANCOVA test adjusted for baseline HDL-C, fat, and carbohydrate

**Table 4:** Biochemical assessment of the intervention (n = 25) and control (n = 25) groups before and after the study, as well as their differences

		Before	After	P-value^#^	Mean difference
Serum zinc (mg/dl)	Intervention	82.92 ± 9.97	86.08 ± 9.43	< 0.001	3.16 ± 1.14
	Control	82.88 ± 9.57	82.80 ± 10.04	0.723	-0.08 ± 1.11
	P-value^*^	0.989	0.240		< 0.001^†^
ALT (IU/L)	Intervention	38.68 ± 35.55	30.36 ± 25.87	0.013	-8.32 ± 15.46
	Control	31.60 ± 23.40	28.04 ± 18.45	0.296	-3.56 ± 16.65
	P-value^*^	0.410	0.717		0.116^†^
AST (IU/L)	Intervention	28.92 ± 17.18	21.16 ± 8.74	0.030	-7.76 ± 16.84
	Control	27.92 ± 15.99	26.40 ± 14.88	0.351	-1.52 ± 7.98
	P-value^*^	0.832	0.136		0.033^†^
FBS (mg/dl)	Intervention	105.04 ± 30.59	91.20 ± 24.07	0.033	-13.84 ± 30.66
	Control	95.08 ± 17.51	86.56 ± 16.85	0.144	-8.52 ± 28.22
	P-value^*^	0.166	0.434		0.402^†^
Insulin (µIU/ml)	Intervention	22.98 ± 16.47	18.77 ± 15.36	< 0.001	-4.20 ± 4.33
	Control	24.70 ± 19.71	23.71 ± 16.69	0.627	-0.99 ± 10.06
	P-value^*^	0.739	0.282		0.326^†^
HOMA-IR	Intervention	6.07 ± 4.49	4.32 ± 3.71	< 0.001	-1.75 ± 1.88
	Control	6.12 ± 4.87	5.18 ± 3.91	0.191	− 0.93 ± 3.49
	P-value^*^	0.971	0.429		0.397^†^
TC (mg/dl)	Intervention	187.92 ± 32.25	168.48 ± 27.60	0.003	-19.44 ± 28.85
	Control	188.80 ± 38.13	187.40 ± 39.42	0.868	-1.40 ± 41.76
	P-value^*^	0.930	0.055		0.045^†^
LDL-C (mg/dl)	Intervention	107.96 ± 22.86	85.40 ± 19.56	< 0.001	-22.56 ± 23.40
	Control	112.16 ± 26.87	110.08 ± 36.92	0.767	-2.08 ± 34.67
	P-value^*^	0.555	0.005		0.014^†^
HDL-C (mg/dl)	Intervention	36.60 ± 7.56	43.96 ± 12.69	0.019	7.36 ± 14.65
	Control	41.72 ± 7.11	43.92 ± 9.16	0.129	2.20 ± 7.00
	P-value^*^	0.017	0.990		0.510^†^
TG (mg/dl)	Intervention	185.88 ± 79.64	152.84 ± 60.41	0.001	-33.04 ± 46.01
	Control	176.12 ± 85.57	174.44 ± 78.66	0.882	-1.68 ± 56.18
	P-value^*^	0.678	0.282		0.082^†^
hs-CRP (ng/ml)	Intervention	4.04 ± 0.87	4.06 ± 0.87	0.888	0.02 ± 0.85
	Control	3.93 ± 0.63	3.92 ± 0.77	0.943	-0.01 ± 0.88
	P-value^*^	0.630	0.549		0.862^†^
MDA (µmol/L)	intervention	3.46 ± 1.56	3.33 ± 0.62	0.715	-0.12 ± 1.67
	control	3.05 ± 0.42	2.92 ± 0.44	0.272	-0.13 ± 0.59
	P value^*^	0.221	0.009		0.930^†^
TAC (µmol/L)	intervention	1.46 ± 0.312	1.48 ± 0.44	0.826	0.02 ± 0.45
	control	1.47 ± 0.267	1.49 ± 0.33	0.795	0.02 ± 0.38
	p-value^*^	0.875	0.911		0.099^†^

ALT: alanine transaminase; AST: aspartate transaminase; FBS: fasting blood sugar; HOMA-IR: homeostatic model assessment for insulin resistance; TC: total cholesterol; LDL-C: low-density lipoprotein; HDL-C: high-density lipoprotein; TG: triglyceride; hs-CRP: high-sensitivity C-reactive protein; MDA: malondialdehyde; TAC: total antioxidant capacity; IU: international unit

Data are reported as Mean ± SD.

* Independent Sample t-test

^#^ Paired t-test

^†^ ANCOVA test adjusted for baseline HDL-C, fat, and carbohydrate

P-value less than 0.05 considered significant

## Discussion

This 8-week clinical trial investigated the effects of Zn supplementation on the Zn status, liver aminotransferases, anthropometric variables, glycemic indices, lipid profile, and some inflammation and oxidative stress parameters in patients with NAFLD. According to the between-group comparison, consuming 30 mg/d Zn had a significant effect on the Zn status, anthropometric parameters, AST, TC, and LDL-C, but no significant effect on ALT, FBS, insulin, HOMA-IR, HDL-C, TG, hs-CRP, MDA, and TAC.

### The effects of Zn on the serum zn

The liver is the storage organ for Zn, and the relationship between Zn and the liver is a cooperative pathway. Zn deficiency is common in NAFLD patients and also causes liver dysfunction [12]. In this study, the mean serum Zn in the intervention group increased significantly. This value decreased in the placebo group, but this decrease was not statistically significant. In general, the mean changes in the serum Zn in the intervention group compared to the placebo were statistically significant. Consistent with our study, receiving Zn supplement caused a significant increase in the serum Zn in obese patients with NAFLD in the previous study [12]. Also, based on the results of previous studies on various diseases such as DM [11], metabolic syndrome [13], obesity [10], and polycystic ovary syndrome [14], Zn supplementation increased the serum Zn in the intervention group, which is consistent with the present study. Factors and mechanisms that reduce Zn in patients with NAFLD include inflammation, oxidative stress, obesity, hypertension, DM, IR, impaired gastrointestinal absorption of Zn, and decreased Zn intake [15].

### The effects of Zn on the liver aminotransferases

Liver enzymes such as ALT and AST were evaluated as markers of NAFLD evaluation in this study. According to the results, Zn supplementation significantly reduced AST, but it had no effect on ALT in NAFLD patients. Furthermore, the results of the study of Fathi et al. were in line with those of our study [12]. The results of a study by Murakami et al. showed that the decrease in ALT due to Zn intake in patients with hepatitis C might be due to the antioxidant role of this element [16]. However, in the study of Idowu et al., the mean of ALT and AST enzymes in the group that received Zn supplementation was higher than the control group [17]. Although Zn is expected to have a beneficial effect on liver enzymes, some conflicting results might be due to long-term and high-dose use as some studies have shown that high doses can be harmful to the hepatocyte membrane by increasing ALT and AST [18].

### The effects of Zn on the anthropometric parameters

Within-group and between-group comparisons indicated a significant decrease in weight, waist circumference, and BMI parameters in the present study. Consistent with our study, the results of previous studies indicated that Zn supplementation improved anthropometric parameters [19–21]. However, the effect of the Zn supplementation on waist circumference was not significant in some studies [13, 14]. Differences in the intervention dose, age group, studied disease, and baseline of mean serum Zn could be the reasons for some inconsistencies with the results of the present study. Zn can improve anthropometric indices by improving the IR index, having insulin-like effects, and enhancing lipid-related metabolic pathways. Zn also increases leptin, which can affect and reduce food intake by producing or inhibiting the production of some appetite mediators. In addition to affecting food intake, leptin increases energy expenditure by stimulating the sympathetic nerves. Besides, leptin rises the expression of the UCP-1 protein-producing gene by stimulating brown adipose tissue and ultimately increases the exothermic effect. Leptin can also increase insulin sensitivity and reduce TG accumulation in the skeletal muscle and liver tissue, regardless of its effect on diet and weight [22–24].

### The effects of Zn on the lipid profile

Dyslipidemia is a common disorder in NAFLD patients that increases the risk of CVD. Based on the results of the present study, the improvement of lipid profile was significant in the intervention group, but not in the control group. Between-group changes were also significant for TC and LDL-C, but not for HDL-C and TG. In the same line with the present study, Zn supplementation in some studies led to significant effects on the lipid profile [8, 25]. Conversely, for some components of the lipid profile, the results of some studies were not consistent with those of the present study [11, 26]. Different doses and duration of intervention may be the main reasons for inconsistent results in our study. Zn has insulin-like effects in the body that can enhance lipogenesis and glucose transport into the cell. As a result, this function can improve the lipid profile and proper function of cells [25, 27, 28]. Another possible mechanism is related to the effect of Zn supplementation on leptin production. Leptin directly improves the lipid profile by increasing insulin sensitivity and reducing TG accretion in the skeletal muscle and liver tissue. Leptin also indirectly improves the lipid profile by reducing food intake and weight [22–24].

### The effects of Zn on the glycemic index

Although Zn supplementation significantly reduced glycemic index (FBS, insulin, and HOMA-IR) in the intervention group, no significant difference was observed in the mean changes of these parameters between the two study groups. Consistent with the present study, the levels of IR [7, 13] and insulin [7] in the intervention group were significantly reduced in previous studies. Unlike the present study, the decrease in insulin, FBS, and IR in the intervention group of the previous study was not significant [29], and insulin levels and IR increased in the placebo group [7]. Zn is an essential element for the processing, storage, and secretion of insulin in the pancreatic beta cells, which are significant reservoirs of Zn. On the other hand, Zn has an insulin-like effect on the body’s cells and can have similar effects even without the presence and function of insulin in the body [28, 30]. The effect of Zn on insulin signaling is related to the stimulation of several compounds including phosphoinositide-3 kinase, phosphorylation of tyrosine from insulin receptor β subunit, and phosphorylation of tyrosine in insulin receptor substrate-1 (IRS-1), and phosphorylation of serine-473 in AKT [28, 31–33]. Zn also indirectly affects the insulin-like growth factor (IGF) via its insulin-like function. Other insulin-like mechanisms of Zn include the inactivation of the glycogen synthase kinase-3β (GSK-3β) enzyme. This enzyme is a protein kinase related to IR, and Zn inactivates GSK-3β by the PI3 / AKT signaling pathway. Moreover, Zn has beneficial effects on IR due to leptin. Various studies have shown that leptin depletion can be considered a factor in increasing IR as described in the previous part [22, 23].

### The effects of Zn on the hs-CRP

Inflammation causes damage to the hepatocytes and the progression of liver disease [34]. Based on the results of the present study, consuming the Zn supplement had no significant effect on the hs-CRP level. Although the results of some studies were in line with those of our study [10, 14], some studies showed inconsistent results and Zn intake reduced the level of hs-CRP [13, 35, 36]. Gut-liver axis can play an effective role in liver disease and increase inflammatory factors [37]. According to studies, Zn supplementation can have positive effects on the gut-liver axis by reducing endotoxemia, reducing oxidative stress and the production of inflammatory cytokines, stabilizing the intestinal defense barrier, and exerting positive effects on hepatocyte apoptosis [38, 39]. Foster and Samman in their study found that Zn supplementation in higher doses equal to 45 mg/d can reduce pro-inflammatory factors. However, at low doses, the effects were different, so even at doses less than 10 mg/d, they had the opposite effect and increased the inflammatory mediators. Consequently, they reported a dose-dependent response of inflammatory factors to the Zn supplementation [40]. As a result, it can be said that one of the possible reasons for the insignificancy of Zn supplementation on inflammatory factors and also discrepancies in the results of different studies in comparison with our research could be the selected dose of 30 mg/d in our study and different doses in other studies.

### The effects of Zn on the oxidative stress

MDA as a marker of fat oxidation and TAC are components of oxidative stress that play an important role in NAFLD. Although we observed a decrease in the MDA level and an increase in TAC in this study, these changes were not statistically significant. While some previous studies were in line with the present study and Zn supplementation did not have a significant effect on MDA [36, 41], in some studies, this factor was significantly reduced [13, 14]. Regarding the TAC, the results of a previous study were similar to those of our study [14], while in some other studies a significant increase in TAC was observed by means of Zn intake [10, 36]. Zn increases the antioxidant activation of proteins, molecules, and enzymes such as glutathione, catalase, and superoxide dismutase and reduces the activity of oxidant-promoting enzymes such as nitric acid synthetase [42, 43]. The difference in the duration of the intervention, the dose of supplementation with Zn as well as the serum Zn level in the studies are likely to be the possible reasons for not relevant effect of Zn supplementation on oxidative stress factors.

### Strengths and limitations of the study

Measuring serum Zn and controlling the confounding effect of food intake by prescribing both groups a low-calorie diet were the strengths of this study. The short duration of the study and small sample size were the limitations of this study. Furthermore, the results of this study cannot be generalized to the entire society and these results should be assessed by future studies.

## Conclusion

Taking 30 mg/d of Zn supplement in addition to a balanced, energy-restricted diet for 8 weeks may increase the Zn serum level and decrease the weight, BMI, waist circumference, AST, TC, and LDL-C in NAFLD patients, so further studies are recommended in the future.

## Data Availability

The datasets used and analyzed during the current study are available from the corresponding author upon reasonable request.
